# Benefits from the first year of GnRHa therapy in boys with idiopathic central precocious puberty when initiating treatment after age 9 years: findings from a real-world retrospective study

**DOI:** 10.1186/s12902-022-01207-z

**Published:** 2022-12-02

**Authors:** Ming-ming Ni, Shu-ting Yang, Wen-wen Wu, Shan-shan Wang, Man Li, Qing-qing Liu, Xing Ji

**Affiliations:** 1 Department of Pharmacy, Children’s Hospital of Nanjing Medical University, 72 Guangzhou Rd, Nanjing, 210008 People’s Republic of China; 2 School of Pharmacy, Nanjing Medical University, Nanjing, 210029 China; 3 Nanjing Medical University, Nanjing, China

**Keywords:** Idiopathic central precious puberty, Gonadotrophin-releasing hormone analog, Final adult height, Predicted adult height, Bone age

## Abstract

**Background:**

GnRHa treatment was established for improving final adult height (FAH) in children presenting with Idiopathic central precocious puberty (ICPP) up to age 8, while several controversies remained for older age groups. The primary objective was to evaluate whether boys diagnosed with ICPP over 9 years of chronological age (CA) could achieve a height benefit from GnRHa treatment.

**Methods:**

We retrospectively evaluated the medical records of 23 boys treated for idiopathic central precocious puberty between January 2018 and January 2021 at Jiangsu Children’s Medical Center. All patients started treatment with intramuscular depot GnRHa at a dose of 80–100 μg/kg, followed by continuous intramuscular injection every 28 days at a dose of 60–80 μg/kg. The hormonal parameters, bone age/chronological age ratio, FAH, growth velocity (GV), tanner staging and body mass index (BMI) were assessed during the treatment period.

**Results:**

After one course of treatment (3 months), the basal FSH and testosterone levels were reduced, while the basal LH value was not significantly changed compared with those before treatment. Furthermore, the mean BA/CA ratio reduction was statistically significant at month 12. The mean PAH following administration of GnRHa after 12 months was statistically improved compared with those at baseline. In addition, the clinical sign of puberty and GV were significantly improved and the BMI remained unchanged as desired at month 12.

**Conclusions:**

This analysis highlighted the positive outcome on the decrease in the rate of bone maturation, with a favorable effect on progression of clinical signs of puberty. Furthermore, our study confirmed PAH was improved even in the older children at onset of treatment (ages 9–10), emphasizing the importance of personalized treatment in such population.

## Introduction

Idiopathic central precocious puberty (ICPP) refers to conditions where disruption of the hypothalamic-pituitary-gonadal (HPG) axis leads to the development of secondary sexual characteristics before age 8 years in girls and 9 years in boys, without evidence of organic lesions in the central nervous system or genetic defects [1]. Early puberty tends to exhibit temporarily rapid acceleration in growth, resulting in early fusion of the epiphyseal growth plates of long bones that causes a decrease in final adult height (FAH) [2, 3]. In addition, rapid progression of the secondary sexual characteristics in children can also cause poor psychological stress, social adaptability, and emotional disorders because of physical differences with peers who have normal puberty [4, 5].

Gonadotrophin-releasing hormone analog (GnRHa) is the preferred drug modelled based on human hypothalamic gonadotropin-releasing hormone (GnRH) for considering intervention of ICPP [6], which downregulate and desensitize the pituitary GnRH receptors, inhibiting the secretion of gonadotropins and sex steroids, thereby slowing the onset of puberty [7]. Clinically, it was expected that long-term GnRHa treatment would lead to substantial improvement of compromised FAH. In fact, GnRHa is able to stabilize pubertal signs, suppress the secretion of sex hormones, decrease the rate of bone maturation and restore growth potential, at least as has been demonstrated in girls [2]. However, as the prevalence of ICPP in boys is less common than that in girls, very little data is available on efficacy of the GnRHa treatment in boys, especially those presenting with ICPP over age 9. It has been suggested that continuing treatment beyond 12 years of bone age (BA) has no further benefit in height outcomes [8–12], and several controversies remain on whether there is benefit from treatment onset after 8 years of chronological age(CA) [13–16]. To begin to fill this knowledge gap, the current study was to evaluate whether boys diagnosed with ICPP over 9 years of CA could achieve a height benefit from GnRHa treatment.

## Materials and methods

### Subjects

This retrospective study was done reviewing the clinical charts of 23 boys with ICPP at Jiangsu Children’s Medical Center between January 2018 and January 2021. All patients started treatment with intramuscular depot GnRHa (Triptorelin acetate, Ipsen Pharma Biotech, France, import registration certificate H20140298) at a dose of 80–100 μg/kg, followed by continuous intramuscular injection every 28 days at a dose of 60–80 μg/kg. No patient received additional medications.

Only those who met all the inclusion criteria were selected for study. Inclusion criteria: (1) testicular volume of 4 ml or more before age 10 years, [17, 18] (2) CA ≥ 9 year and advancement of BA over CA at the time of diagnosis (3) The children had a pubertal LH response 60 min after triptorelin acetate stimulation defined as LH   ≥  8 IU/L before treatment initiation, and LH peak/follicle-stimulating hormone(FSH) peak greater than 0.6 in the GnRHa stimulation test using immunochemiluminescence assay [18] (4) brain magnetic resonance imaging was performed to rule out central nervous system tumors. Exclusion criterion: (1) demonstrable organic diseases that could affect HPG axis function, including a thalamus, pituitary tumor and other central nervous system diseases. (2) blood and imaging evaluation of liver, renal, thyroid, adrenal, and pituitary diseases showed peripheral or organic CPP or other types of precocious puberty.

All procedures performed in this study involving human participants were in accordance with the Declaration of Helsinki. This study was approved by the Ethics Committee of the Children’s Hospital of Nanjing Medical University. And the need for informed consent was waived by Ethics Committee of the Children’s Hospital of Nanjing Medical University, because of the retrospective nature of the study.

### Methods

Physical examinations were conducted by a professionally trained pediatric endocrinologist. Pubertal development was assessed according to Tanner [19].BA was measured using the Greulich-Pyle method by taking simple radiographs of the left hand and wrist [20]. Baseline growth rate was calculated from two measurements of height at least 12 months apart. GnRH stimulation tests were performed to evaluate pubertal response in all subjects. Predicted adult height (PAH) was calculated by dividing a boy’s height by the average percentage of mature height associated with a given BA based on tables published by Li et al. [21], which has a better fit with the FAH in Chinese population compared to Bayley–Pinneau method [22].

### Measures

Data were obtained from a retrospective review of the medical records. Bathe initial evaluation included determination of BA, height, weight, pubertal stage, testicular volume, growth velocity (GV) and evaluation of the HPG axis by measuring basal and peak levels of LH and FSH and the plasma concentrations of testosterone. After 3 months of therapy, the hormone testing was repeated in study population to evaluate effective suppression of the HPG axis.BA and PAH were determined at baseline, 6 and 12 months. Growth velocity was calculated using height at initial evaluation and follow-up 12 months with treatment.

Patients’ sexual development symptoms, the auxological parameters including patient height and weight were monitored and recorded before and after treatment. During the treatment, we monitored blood pressure, heart rate, and other vital signs. We also regularly examined our patients to detect any possible adverse drug reactions or local reactions, headaches, fatigue, flushed face, and other serious complications.

## Statistical analysis

Results are presented as means ± SD. The Student’s t-test was used for the comparison of the means of the two samples that conformed to the normal distribution and the homogeneity of variance, and the paired Student’s t-test was used for the pairwise comparison of the same subject at different time points. The Mann–Whitney test was used for two-group comparisons of non-normally distributed data, and the χ^2^ test was used to compare the constituent ratios in the enumeration data. A *p*-value of 0.05 was considered statistically significant. Data obtained were analyzed using the Statistical Package for the Social Sciences, PSS ver. 15.0 (SPSS Inc., Chicago, IL, USA).

## Results

### Baseline characteristics of the study subject

A total of 23 boys with ICPP were enrolled in this study. The demographic and disease characteristics in terms of CA, weight, height, BMI, pubertal stage, GV, BA and PAH are summarized in Table 1.

**Table 1:** Subject characteristics at baseline

Characteristic	Baseline (*n* = 23)
Chronological age, mean (SD), years	9.67 ± 0.42
Weigh, Mean (SD), kg	43.83 ± 7.84
Height, mean (SD), cm	147.10 ± 4.62
BMI, (SD), kg/m^2^	20.17 ± 3.06
Testicular volume, Mean (SD), ml	10.39 ± 4.11
Growth velocity, mean (SD), cm/year	10.46 ± 2.26
Bone age, Mean (SD), years	12.40 ± 1.09
Bone age/Chronological age, mean (SD)	1.28 ± 0.11
Predicted adult height, Mean (SD), cm	163.70 ± 5.77

### Hormonal parameters

After one course of treatment (3 months), the basal FSH level decreased from 3.75 ± 1.18 IU/L at baseline to 0.98 ± 0.65 IU/L at month 3 after GnRHa treatment. Similarly, the mean testosterone (TE) also sharply regressed from 5.19 ± 4.41 nmol/L at baseline to 0.16 ± 0.10 nmol/L at month 3. And all subjects sustained suppression of levels FSH and testosterone for the duration of the study. Conversely, a plunge of LH in response to GnRHa treatment was not detected in 13 patients, occurring in 56.5% of children, despite every other parameter indicated pubertal suppression. And the mean basal LH value following administration of GnRHa after 3 months was not significantly changed compared with those before treatment (2.09 ± 0.85 IU/L versus 2.80 ± 1.64 IU/L, respectively, P>0.05). The mean LH and FSH levels as well as the testosterone levels throughout the study are illustrated in Table 2.

**Table 2:** Changes in the basal serum luteinizing hormone (LH), follicle-stimulating hormone (FSH) and testosterone (TE) levels from baseline to one course of treatment

Time point, month	LH, IU/L	FSH, IU/L	TE, nmol/L
0	2.09 ± 0.85	3.75 ± 1.18	5.19 ± 4.41
3	2.80 ± 1.64	0.98 ± 0.65	0.16 ± 0.10
*P* value	> 0.05	< 0.05	< 0.05

### Bone age/chronological age ratio

**Fig. 1:**
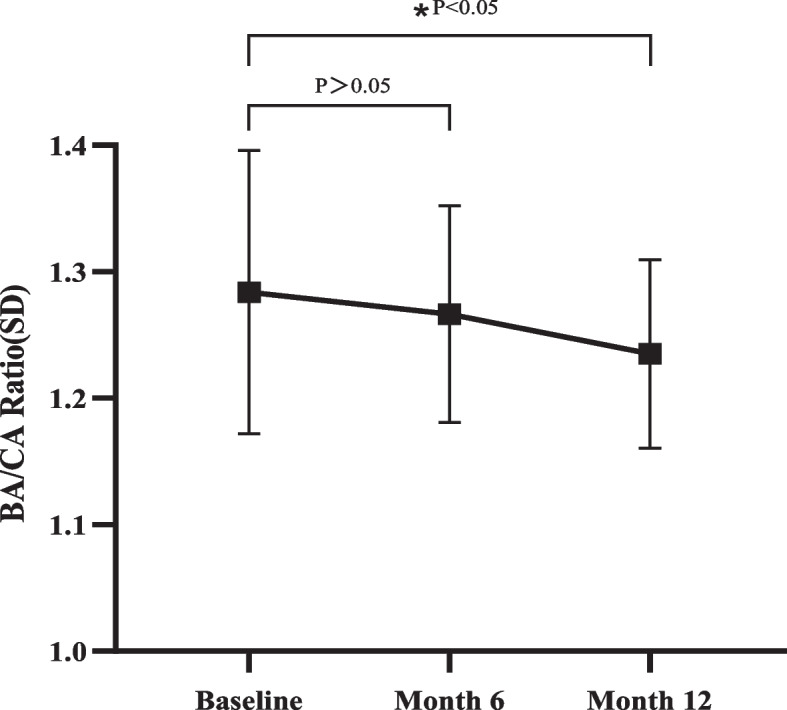
Change in mean BA/CA ratio at baseline, month 6 and month 12

The CA and BA at the time of diagnosis was 9.67 ± 0.42 years and 12.40 ± 1.09 years, respectively. The CA was 10.17 ± 0.42 years and the BA was 12.85 ± 0.61 years following administration of GnRHa after 6 months. After 1 year of treatment, the CA was 10.67 ± 0.42 years, and the BA was 13.15 ± 0.59 years (Table 3). The percentage of children with reduction in BA/CA ratio was 78.3% at month 6 and 82.6% at month 12. Furthermore, the mean BA/CA ratio reduction was statistically significant from 1.28 ± 0.11 at baseline to 1.24 ± 0.074 at month 12, suggesting that bone maturation inhibition was positively associated with duration of treatment (Fig. 1). BA-CA difference decreased from 2.74 ± 1.04 at baseline to 2.49 ± 0.73 at month 12 during the treatment period, but there was no statistical significance.

**Table 3:** Changes in Chronological age (CA), Bone age (BA) and BA/CA ratio after 6 and 12 months of GnRHa therapy

Time point, month	CA, years	BA, years	BA/CA ratio
0	9.67 ± 0.42	12.40 ± 1.09	1.28 ± 0.11
6	10.17 ± 0.42	12.85 ± 0.61	1.27 ± 0.086^#^
12	10.67 ± 0.42	13.15 ± 0.59	1.24 ± 0.074*

*P* value, *P*^#^ > 0.05, P* < 0.05

### Predicted adult height

**Fig. 2:**
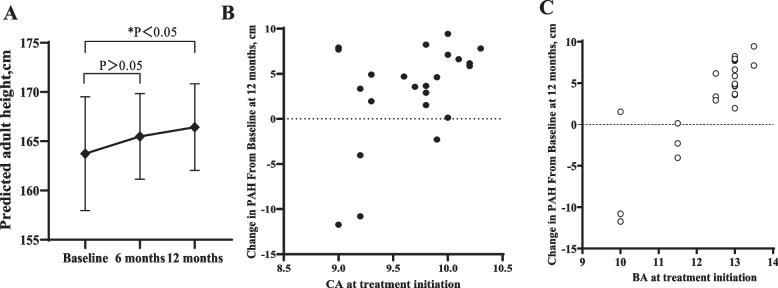
Change in predicted adult height by duration of treatment (**A**), chronological age at start of treatment (**B**), and bone age (**C**)

The mean PAH following administration of GnRHa after 12 months was statistically improved compared with those at baseline (163.76 ± 5.76 cm versus 166.76 ± 4.22, respectively, *P* < 0.05)(Fig. 2A). And the majority of boys (18/23) regardless of BA at treatment initiation showed improvement in PAH with longer duration of treatment averaged 5.16 ± 2.57 (range from 1.52 to 9.43) cm, suggesting the pre-specified primary efficacy endpoint was achieved. Nine of the boys evaluated in one-year experienced PAH improvements > 5 cm, an arbitrary threshold for clinical significance, while one of the boys remained unchanged in PAH during that year. Interestingly, 4 of the boys whose PAH decreased (− 2.3 to − 11.7 cm) had lower BAs at the start of treatment, indicating that it may not be possible to predict the response based on the initial bone age (Fig. 2B and C).

### Other observation indexes

Growth velocity declined from a mean baseline value of 10.46 ± 2.26 cm/year at baseline to 7.40 ± 1.04 cm/year at month 12, suggesting that growth acceleration was slowing down. This, in turn, reduces the GV, giving the long bones more time to lengthen before the grow plates fuse, thus maximizing the adult height that the child obtains. There were at least 19 patients out of 23 showed absences of progression of testis volumes at months 12(Data for four boys were not mentioned in the medical record). And the mean body mass index (BMI) was not changed significantly at month 12 compared to baseline (Table 4).

**Table 4:** The Growth velocity(GV) and BMI characteristics at baseline and month 12

Time point, month	GV, cm/year	BMI, kg/m^2^
0	10.46 ± 2.26	20.17 ± 2.97
12	7.40 ± 1.04	20.58 ± 2.78
*P* value	*P* < 0.05	*P* > 0.05

## Discussion

Basal unstimulated LH is alternatively used to monitor the pituitary suppression during GnRHa treatment due to cost-effectiveness, less time consuming and invasive, although peak-stimulated LH levels have been considered the gold standard to therapeutic monitoring [23]. Nevertheless, it is unlikely to be a coincidence that a considerable percentage of basal unstimulated LH concentrations remained at or well above the baseline after one course of treatment in this study. And the persistent elevation or the temporal decline in basal unstimulated LH during subsequent treatments have also been observed. This confirms previous conclusions that elevated concentrations of basal unstimulated LH values fail to demonstrate insufficient pituitary suppression, as other hormonal parameters and non-hormonal parameters suggest apparent pubertal suppression, and are thus unsuitable for routine therapeutic monitoring [24–26]**.**

Suppression of sex steroids caused a progressive decrease in bone maturation, as demonstrated here, the BA/CA ration tapered off over time. Nevertheless, GnRHa did not statistically significant inhibit bone maturation at month 6 because there may not be enough patients whose BA was sufficiently suppressed to be statistically significant, and the precision of GV was not sensitive enough to detect significant changes over a 6-month interval. Consequently, GV was calculated using height at initial evaluation and follow-up 12 months with treatment in present study. Based on the results that we observed in this study, the BA/CA and GV appeared to have decreased as desired at month 12. In addition. It is important to note that the BMI did not change significantly during the study, which is in accordance with previous findings that long-term GnRHa therapy has no detrimental effects on BMI [27, 28]. Furthermore, similarly to other studies, there were no substantial unexpected adverse events were found in this study.

This study addressed specific features of male patients regarding benefit in height outcomes with continued treatment even beyond 9 years of CA or 12 years of BA, suggesting that no absolute CA or BA limitation should dictate the initiation or cessation of treatment. As for deciding on treatment in such cases, treatment regimens should be individually-tailored, and neither treatment initiation nor cessation should be based solely on BA or CA. Of course, the results of this study need to be validated in a larger randomized controlled trial. The sample size included in the study was too small to be fully convincing. Another limitation of this study, common to other ICPP studies, is that the patients were only evaluated in the first year of treatment and were not followed up to FAH, while PAH results were slightly week due to different algorithms.

In conclusion, this analysis highlighted the positive outcome on the decrease in the rate of bone maturation, with a favorable effect on progression of clinical signs of puberty. Furthermore, our study confirmed PAH was improved even in the older children at onset of treatment (age 9–10), emphasizing the importance of personalized treatment in such population.

## Data Availability

The datasets used or analyzed during the current study are not publicly available due to legal and ethical reasons but are available from the corresponding author on reasonable request.

## Abbreviations

ICPP: Idiopathic central precocious puberty
HPG: Hypothalamic-pituitary-gonadal
GnRHa: Gonadotrophin-releasing hormone analog
FAH: Final adult height
PAH: Predicted adult height
BA: Bone age
CA: Chronological age
LH: Luteinizing hormone
FSH: Follicle-stimulating hormone
GV: Growth velocity
BMI: Body mass index
